# Supplementary material for: Prostaglandin E2 controls the metabolic adaptation of T cells to the intestinal microenvironment
Source: Nat Commun. 2024 Jan 11;15:451. doi: 10.1038/s41467-024-44689-2 (PMC10781727; doi:10.1038/s41467-024-44689-2)

**Supplementary information**

Prostaglandin $E_2$ controls the metabolic adaptation of T cells to the intestinal microenvironment

Villa M et al.

## Supplementary figures

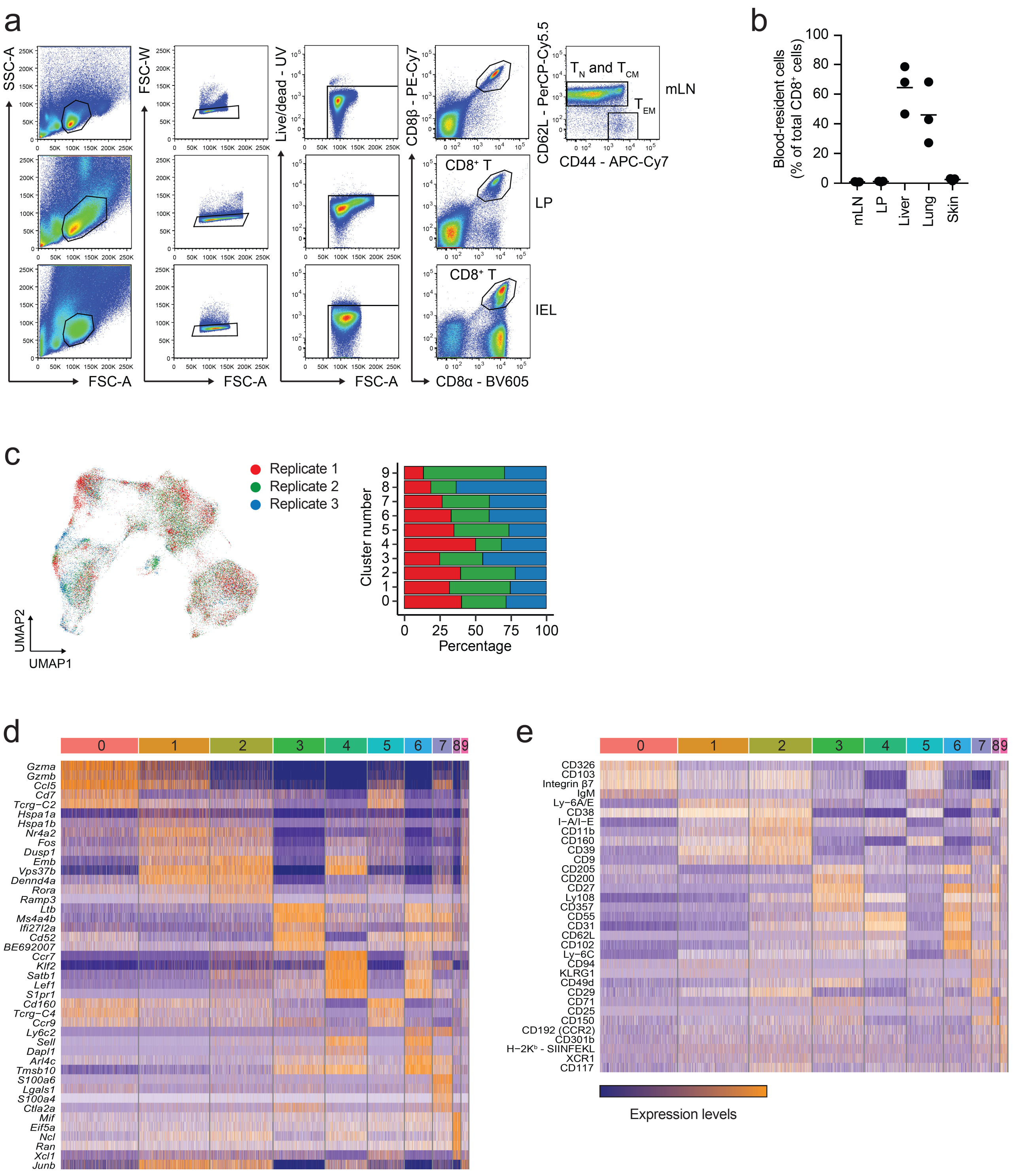


*Supplementary Figure 1. Single cell RNA sequencing reveals the dynamics of the intestinal immune response under homeostatic conditions.*

*a*. Representative flow cytometry plots to show the sorting strategy of the experiments designed in *Figure 1*. Cells were sorted from the $T_N$ and $T_{CM}$ and $T_{EM}$ gates of mLN, $CD8^+$ T gates of LP and IEL. *b*. Dot plot showing the fraction of $CD8^+$ T cells localized in the vasculature at the time of isolation from the indicated tissues. Data were obtained by intravascular staining with anti-CD45 antibody.

Data show n = 3 biological replicates from one experiment.

*c*. UMAP of the single cell RNA and ADT sequencing data obtained from the experiments designed as in *Figure 1*. Color-coding is according to the biological replicate as outlined in the legend. The bar graph shows the distribution of the analyzed cells in every cluster across the biological replicates.

*d*. Heatmap showing the top 5 DEGs in each cluster. Every row in heatmap corresponds to a gene, every column to a cell. Color coding: orange, high expression; purple, low expression.

*e.* Heatmap showing the top 5 ADTs in each cluster. Every row in heatmap corresponds to a surface protein, every column to a cell. Color coding: orange, high expression; purple, low expression.

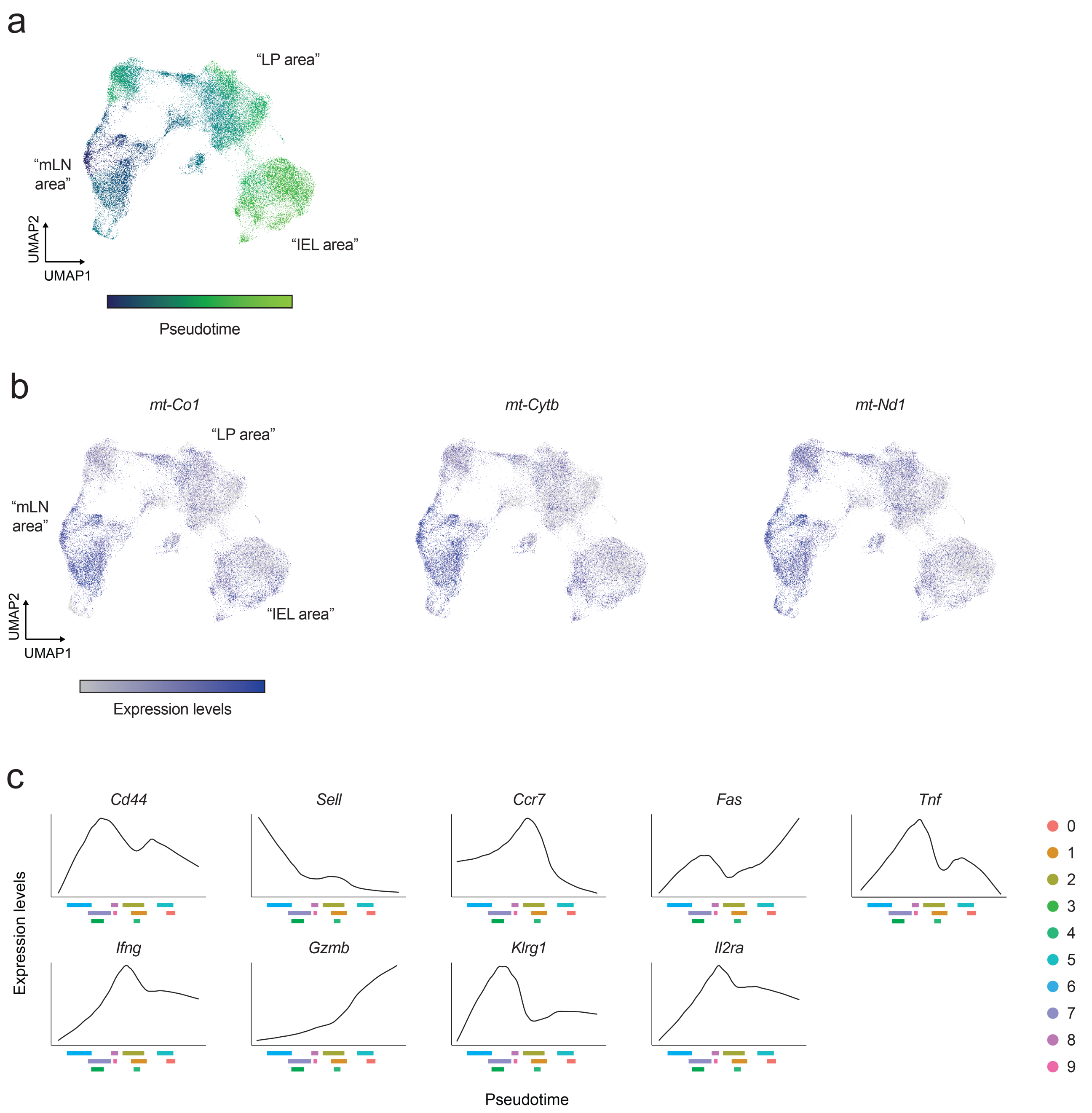


*Supplementary Figure 2. Adaptation of CD8$^{+}$ T cells to the gut microenvironment correlates with transcriptional and surface changes.*

*a*. UMAP of the single cell RNA and ADT sequencing data showing the progressive pseudotime of the gut CD8$^{+}$ T cell response. Color-coding indicates the pseudotime, as indicated in the legend. Cluster 6 was set as the starting point of the analysis. The labeling in quotation marks refers to the data in *Figure 1c*.

*b*. UMAP of the single cell RNA and ADT sequencing data showing expression of selected genes across different clusters. The labeling in quotation marks refers to the data in *figure 1c*.

*c*. Plots representing the expression of the selected indicated genes as a function of pseudotime, as identified in *Supplementary Figure 2a*. On the x axis, the interquartile range of every cluster is

indicated, to facilitate the correlation between gene expression and progression of cells through pseudotime. Color-coding is indicated in the legend.

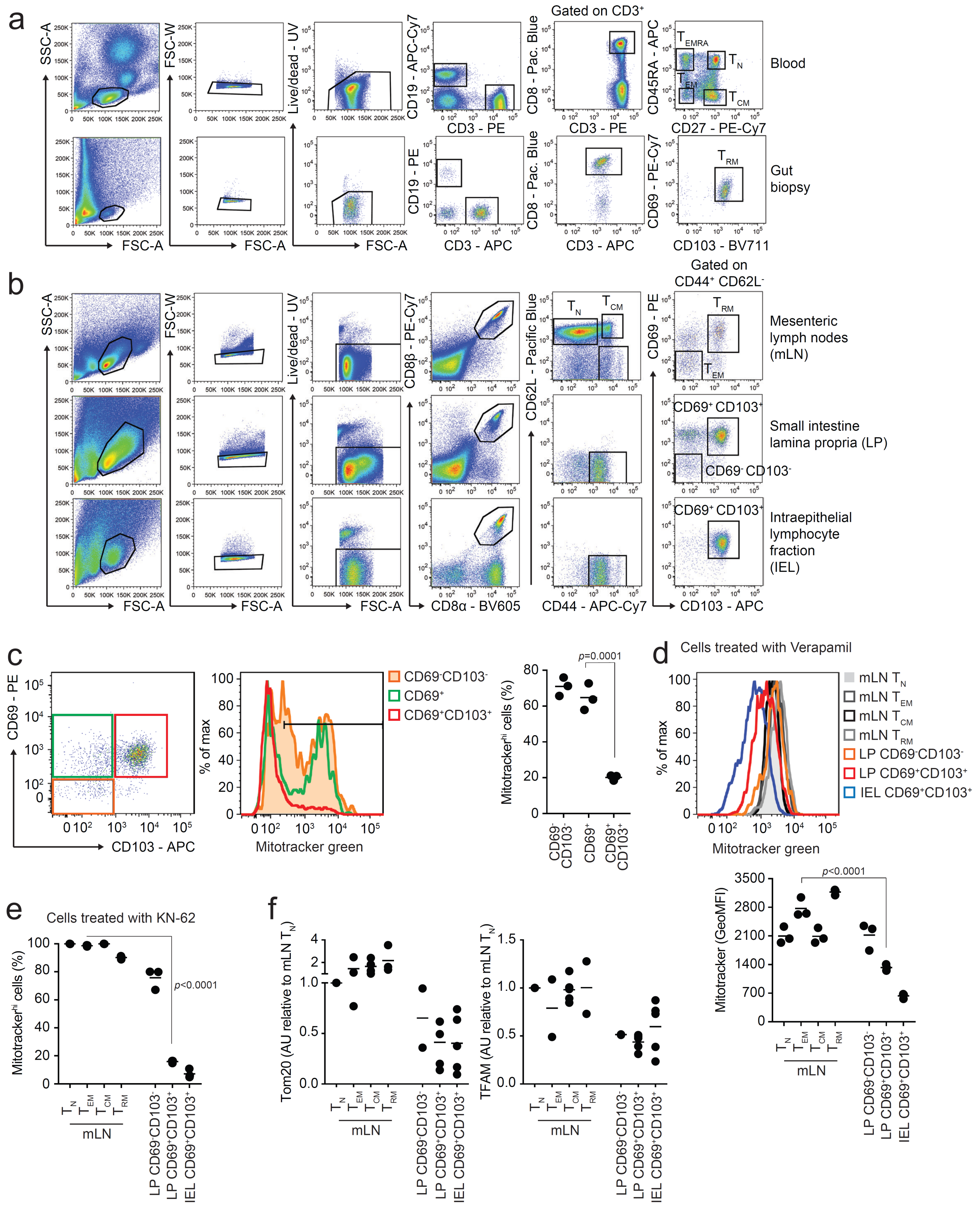

*Supplementary Figure 3. Drop in mitochondrial content underlies the spatial transition of T cells from the lymph nodes into the intestinal compartments.*

*a*. Representative flow cytometry plots to show the gating strategy of the human experiments represented in the paper. Cell populations analyzed were: $T_N$, $T_{EM}$, $T_{CM}$ and $T_{EMRA}$ from the blood;

and $T_{RM}$ from intestinal biopsies.

*b*. Representative flow cytometry plots to show the gating strategy of the mouse experiments represented in the paper. Cell populations analyzed were $T_N$, $T_{EM}$, $T_{CM}$ and $T_{RM}$ from the mLN, $CD69^-CD103^-$ and $CD69^+CD103^+$ cells from LP and $CD69^+CD103^+$ cells from IEL.

*c*. Flow cytometry analysis of Mitotracker green staining in the indicated cell populations across the phenotypes $CD69^-CD103^-$ - $CD69^+CD103^-$ - $CD69^+CD103^+$ cells isolated from LP of unchallenged C57BL/6J mice. Color-coding is as per legend. The flow cytometry plot shows the gating strategy based on CD69 and CD103 expression. Lines in the dot plot show mean values and the data show n = 3 biological replicates over nine independent experiments. Statistics were performed using one-way ANOVA and Tukey's multiple comparison correction.

*d*. Flow cytometry analysis of Mitotracker green staining in the indicated cell populations isolated from mLN and intestine of unchallenged C57BL/6J mice, upon treatment of cells with Verapamil. Color-coding is as per legend. Lines in the dot plot show mean values and the data show n = 3 biological replicates over two independent experiments. Statistics were performed using one-way ANOVA and Tukey's multiple comparison correction.

*e*. Flow cytometry analysis of Mitotracker green staining in the indicated cell populations isolated from mLN and intestine of unchallenged C57BL/6J mice, upon performing cell isolation in presence of KN-62. Lines in the dot plot show mean values and the data show n = 3 biological replicates from one experiment. Statistics were performed using one-way ANOVA and Tukey's multiple comparison correction.

*f*. Western blotting analysis of Tom20 and TFAM in the indicated cell populations. Dot plots show the quantification by densitometry of the levels of Tom20 and TFAM as compared to β-actin, shown in *Figure 2c*. Lines in the dot plot show mean values and the data are cumulative of two to five independent experiments.

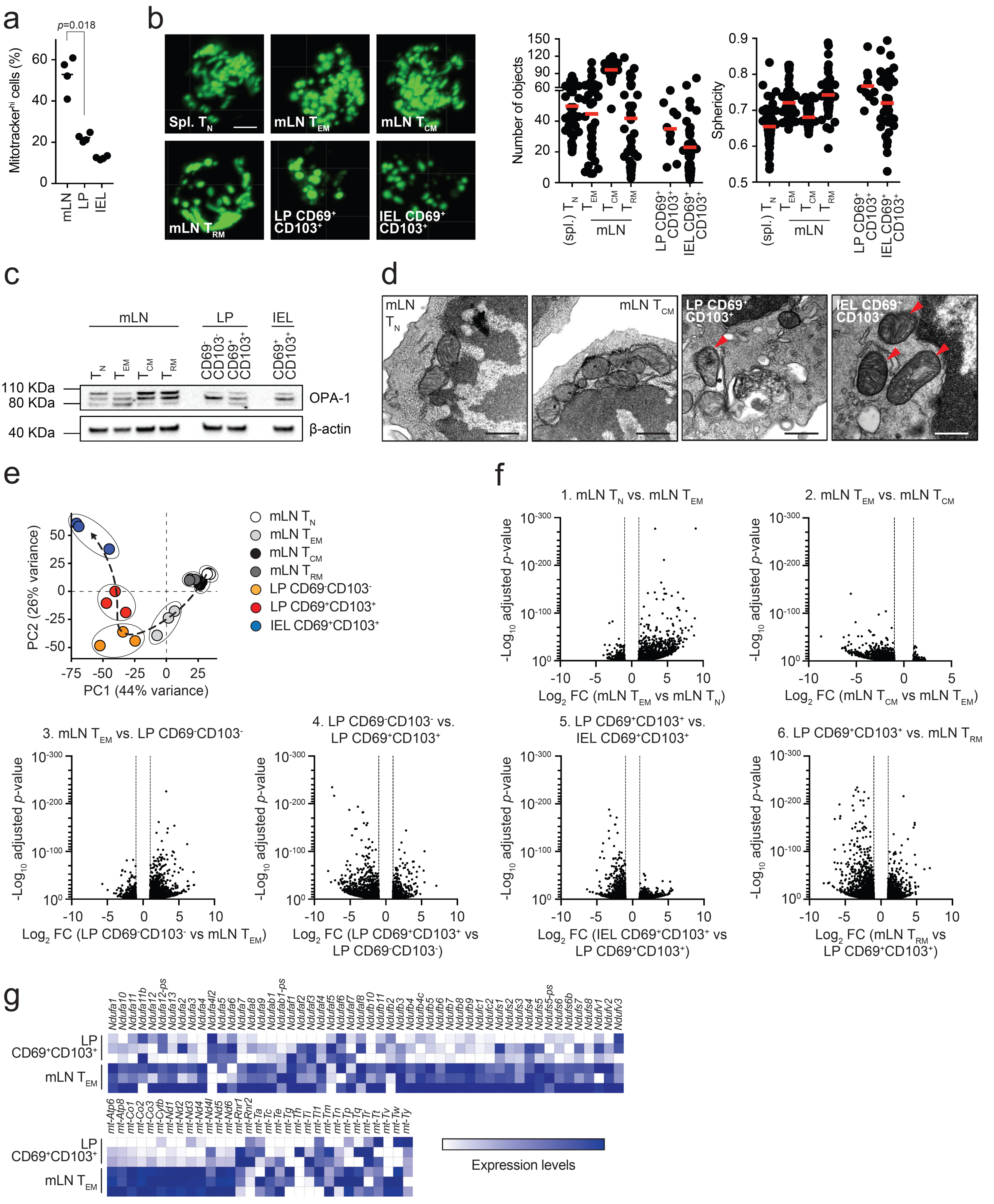

*Supplementary Figure 4. Exposure to the gut environment impacts the structure and function of mitochondria.*

*a*. Flow cytometry analysis of Mitotracker green staining in antigen-specific ($CD45.2^+$) OT-I cells isolated from mLN and intestine of C57BL/6J mice orally challenged with *LmOVA* and analyzed 24 days post-infection. Lines in the dot plot show mean values and data show n = 4 biological replicates

over two independent experiments. Statistics were performed using one-way ANOVA and Tukey's multiple comparison correction.

*b*. Fluorescence microscopy of the indicated cell populations isolated from unchallenged PhAM mice. $T_N$ cells were isolated from the spleen. Each dot represents the number of objects counted per cell. Scale bar = 2 μm. Red lines in the dot plots indicate mean values and the plots show cumulative data of n = 40 cells (10 cells for LP $CD69^+CD103^+$) from two independent experiments.

*c*. Western blotting analysis of OPA-1 in the indicated cell populations. β-actin loading control is the same as the one used in *Figure 2c*. The data shown are representative of six independent experiments.

*d*. Transmission electron microscopy of the indicated cell populations isolated from unchallenged C57BL/6J mice. Red arrowheads indicate mitochondria with dilated cristae. Scale bar = 500 nm. The panel show representative images of one experiment.

*e*. Principal Component Analysis (PCA) of bulk RNA sequencing data obtained from the indicated cell populations. Color-coding is as per legend. Data show three independent experiments.

*f*. Volcano plots showing the distribution according to fold change (FC) and *p*-value of differentially-expressed genes between the indicated cell populations, as obtained upon bulk RNA sequencing. The cellular transitions mentioned in the main text are indicated at the top of every plot. Dashed lines indicate the fold change filter of FC > 2 and FC < -2. Data show three independent experiments.

*g*. Heatmaps showing the expression of selected genes (Complex I of ETC and mitochondria DNA-encoded genes) of bulk RNA sequencing data obtained from the indicated cell populations. Color-coding is as per legend (white, low expression; blue, high expression) and is showing relative values within each gene. Data show three independent experiments.

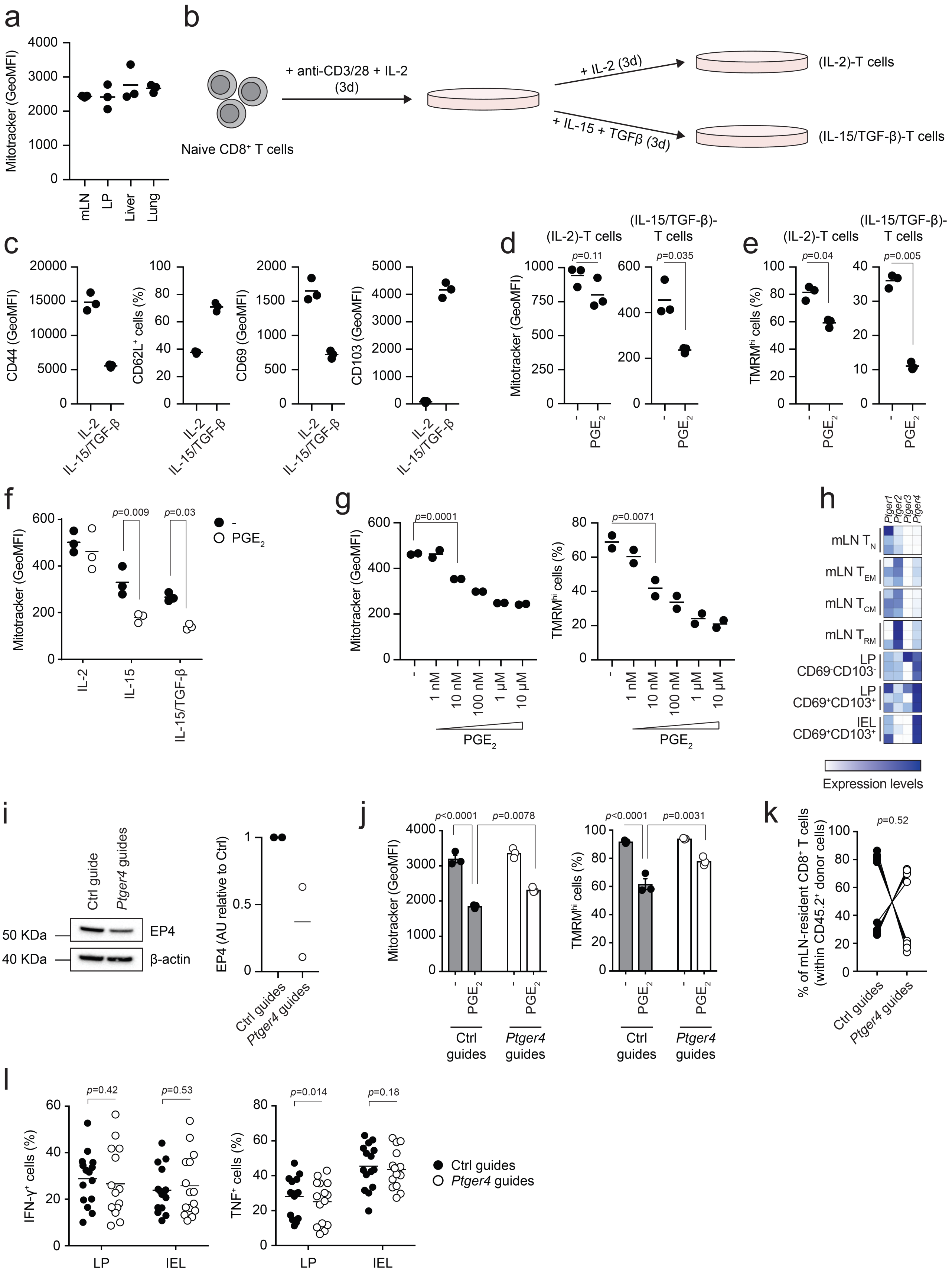
a
b
c
d
e
f
g
h
i
j
k
l
Mitotracker (GeoMFI)
mLN
LP
Liver
Lung
Naive CD8+ T cells
+ anti-CD3/28 + IL-2 (3d)
+ IL-2 (3d)
+ IL-15 + TGFβ (3d)
(IL-2)-T cells
(IL-15/TGF-β)-T cells
CD44 (GeoMFI)
CD62L+ cells (%)
CD69 (GeoMFI)
CD103 (GeoMFI)
IL-2
IL-15/TGF-β
p=0.11
p=0.035
PGE2
TMRMhi cells (%)
p=0.04
p=0.005
p=0.009
p=0.03
IL-15
p=0.0001
p=0.0071
1 nM
10 nM
100 nM
1 μM
10 μM
Ptger1
Ptger2
Ptger3
Ptger4
mLN TN
mLN TEM
mLN TCM
mLN TRM
LP CD69-CD103-
LP CD69+CD103+
IEL CD69+CD103+
Expression levels
Ctrl guide
Ptger4 guides
50 KDa
40 KDa
EP4
β-actin
EP4 (AU relative to Ctrl)
Ctrl guides
p<0.0001
p=0.0078
p=0.0031
% of mLN-resident CD8+ T cells (within CD45.2+ donor cells)
p=0.52
IFN-γ+ cells (%)
TNF+ cells (%)
p=0.42
p=0.53
p=0.014
p=0.18
IEL

*Supplementary Figure 5. The sensing of $PGE_2$ via the EP4 receptor shapes the mitochondrial profile of LP and IEL $CD8^+$ T cells.*

*a*. Flow cytometry analysis of Mitotracker green staining in macrophages isolated from the indicated tissues of unchallenged C57BL/6J mice. Lines in the dot plot show mean values and the data show n = 3 biological replicates over two independent experiments.

*b*. Schematic representation of the *in vitro* cell culture to generate (IL-2)-T cells and (IL-15/TGF-β)-T cells.

*c*. Flow cytometry analysis of the surface expression of the indicated markers in (IL-2)-T cells and (IL-15/TGF-β)-T cells 6 days after activation. Lines in the dot plots show mean values and the data show n = 3 biological replicates over three independent experiments.

*d, e*. Flow cytometry analysis of Mitotracker green (*d*) and TMRM (*e*) staining in $CD8^+$ T cells activated in IL-2- or IL-15/TGF-β-polarizing conditions for 6 days and treated for 24 hours with 100 nM $PGE_2$. Lines in the dot plot show mean values and the data show n = 3 biological replicates over two independent experiments. Statistics were performed using two-tailed Student's *t* test.

*f*. Flow cytometry analysis of Mitotracker green staining in $CD8^+$ T cells activated in IL-2-, IL-15- or IL-15/TGF-β-polarizing conditions for 6 days and treated for 24 hours with 100 nM $PGE_2$. Lines in the dot plot show mean values and the data show n = 3 biological replicates over two independent experiments. Statistics were performed using two-way ANOVA and Sidak's multiple comparison correction.

*g*. Flow cytometry analysis of Mitotracker green and TMRM staining in $CD8^+$ T cells activated in (IL-15/TGF-β)-T cell-polarizing conditions for 5 days and treated for 24 hours with different concentrations of $PGE_2$. Lines in the dot plot show mean values and the data show n = 2 biological replicates over two independent experiments. Statistics were performed using one-way ANOVA and Dunnet's multiple comparison correction.

*h*. Heatmaps show the expression of selected genes of bulk RNA sequencing data obtained from the indicated cell populations. Color-coding is as per legend (white, low expression; blue, high expression) and shows relative values within each gene. Data show three independent experiments.

*i*. Western blotting analysis of EP4 in $CD8^+$ T cells treated for CRISPR-Cas9-mediated deletion of *Ptger4* and activated in (IL-15/TGF-β)-T cell-polarizing conditions for 6 days. Dot plots show the quantification by densitometry of the levels of EP4 as compared to β-actin. Lines in the dot plot show mean values and the data are cumulative of two independent experiments.

*j*. Flow cytometry analysis of Mitotracker green and TMRM staining in $CD8^+$ T cells treated for CRISPR-Cas9-mediated deletion of *Ptger4* and activated in (IL-15/TGF-β)-T cells polarizing conditions for 5 days followed by 24h treatment with 100 nM $PGE_2$. Data show n = 3 biological replicates over two independent experiments. Statistics were performed using one-way ANOVA and Tukey's multiple comparison correction.

*k*. Flow cytometry analysis of the distribution of Ctrl vs *Ptger4*-deleted $CD8^+$ T cells within the

population of CD45.2$^+$ cells upon *LmOVA* challenge in mLN. Lines in the dot plot show pairing within single mice, and the dot plots show cumulative data of n = 10 biological replicates over two independent experiments. Statistics were performed using two-tailed paired *t* test.

*I*. Flow cytometry analysis of interferon-γ and TNF production by Ctrl vs *Ptger4*-deleted CD8$^+$ T cells within the population of CD45.2$^+$ cells isolated from LP and IEL, 7 days after *LmOVA* challenge, upon PMA+ionomycin restimulation for 4h. Lines in the dot plot show mean values; dot plots show cumulative data of n = 15 biological replicates over three independent experiments. Statistics were performed using two-way ANOVA and Sidak's multiple comparison correction.

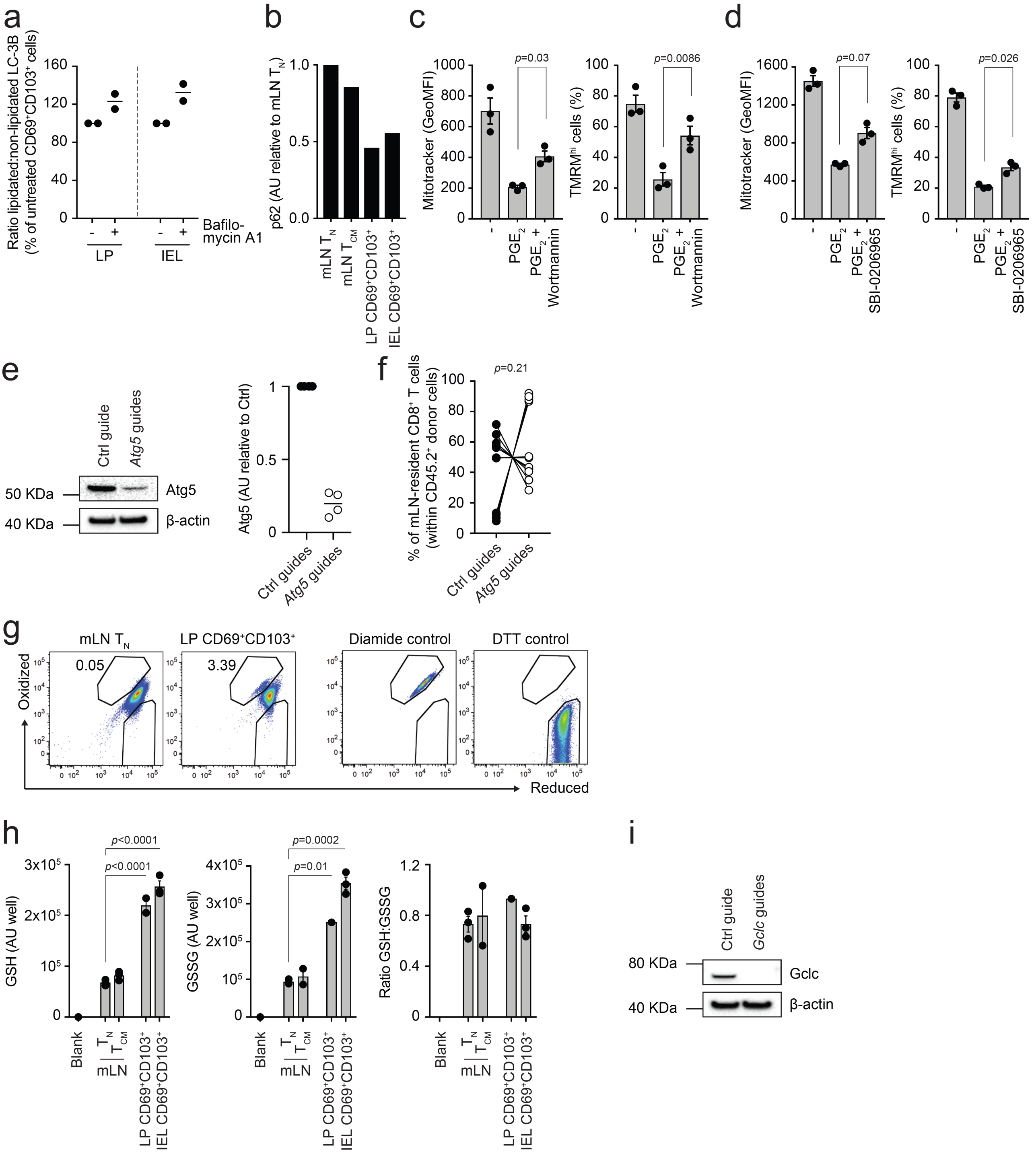


*Supplementary Figure 6. Autophagy and glutathione are key to maintain fitness of intestinal LP and IEL CD8⁺ T cells.*

*a*. Western blot analysis of LC3-I and LC3-II in the indicated cell populations, isolated in presence or absence of Bafilomycin A1. Lines in dot plots show mean values. Dot plots show cumulative data of two independent experiments.

*b*. Western blot analysis of p62 in the indicated cell populations. The bar graph shows the quantification by densitometry of the levels of p62 as compared to β-actin, shown in *Figure 4b*. Data

are from one experiment.

*c*. Flow cytometry analysis of Mitotracker green and TMRM staining in $CD8^+$ T cells activated in (IL-15/TGF-β)-T cell-polarizing conditions for 5 days and treated with $PGE_2$ in presence of wortmannin. Lines in the dot plot show mean values ± SEM and the data show n = 3 biological replicates over three independent experiments. Statistics were performed using one-way ANOVA and Tukey's multiple comparison correction.

*d*. Flow cytometry analysis of Mitotracker green and TMRM staining in $CD8^+$ T cells activated in (IL-15/TGF-β)-T cell-polarizing conditions for 5 days and treated with $PGE_2$ in presence of SBI-0206965. Lines in the dot plot show mean values ± SEM and the data show n = 3 biological replicates from two independent experiments. Statistics were performed using one-way ANOVA and Tukey's multiple comparison correction.

*e*. Western blot analysis of Atg5 in $CD8^+$ T cells treated for CRISPR-Cas9-mediated deletion of *Atg5* and activated in (IL-15/TGF-β)-T cell-polarizing conditions for 6 days. Dot plots show the quantification by densitometry of the levels of Atg5 as compared to β-actin. Lines in the dot plot show mean values and the data are cumulative of four independent experiments.

*f*. Flow cytometry analysis of the distribution of Ctrl vs *Atg5*-deleted $CD8^+$ T cells within the population of $CD45.2^+$ cells upon *LmOVA* challenge in mLN. Lines in the dot plot show pairing within single mice, and the dot plots show cumulative data of n = 13 biological replicates over three independent experiments. Statistics were performed using two-tailed paired *t* test.

*g*. Representative flow cytometry plots to show the gating strategy used to quantify the fraction of cells with oxidized mitochondria, in Mito-roGFP2-Orp1 mice, as shown in *Figure 4h*.

*h*. Biochemical quantification of the concentrations of GSH and GSSG, and their ratio, in the indicated cell populations. The bar graphs show concentrations of GSH and GSSG as arbitrary units (AU) per well containing $10^5$ sorted cells. The bars show mean ± SEM. Data are representative of three independent experiments. Statistics were performed using one-way ANOVA and Tukey's multiple comparison correction.

*i*. Western blot analysis of Gclc in $CD8^+$ T cells treated for CRISPR-Cas9-mediated deletion of *Gclc* and activated in (IL-15/TGF-β)-T cell-polarizing conditions for 6 days. Data are from one experiment.

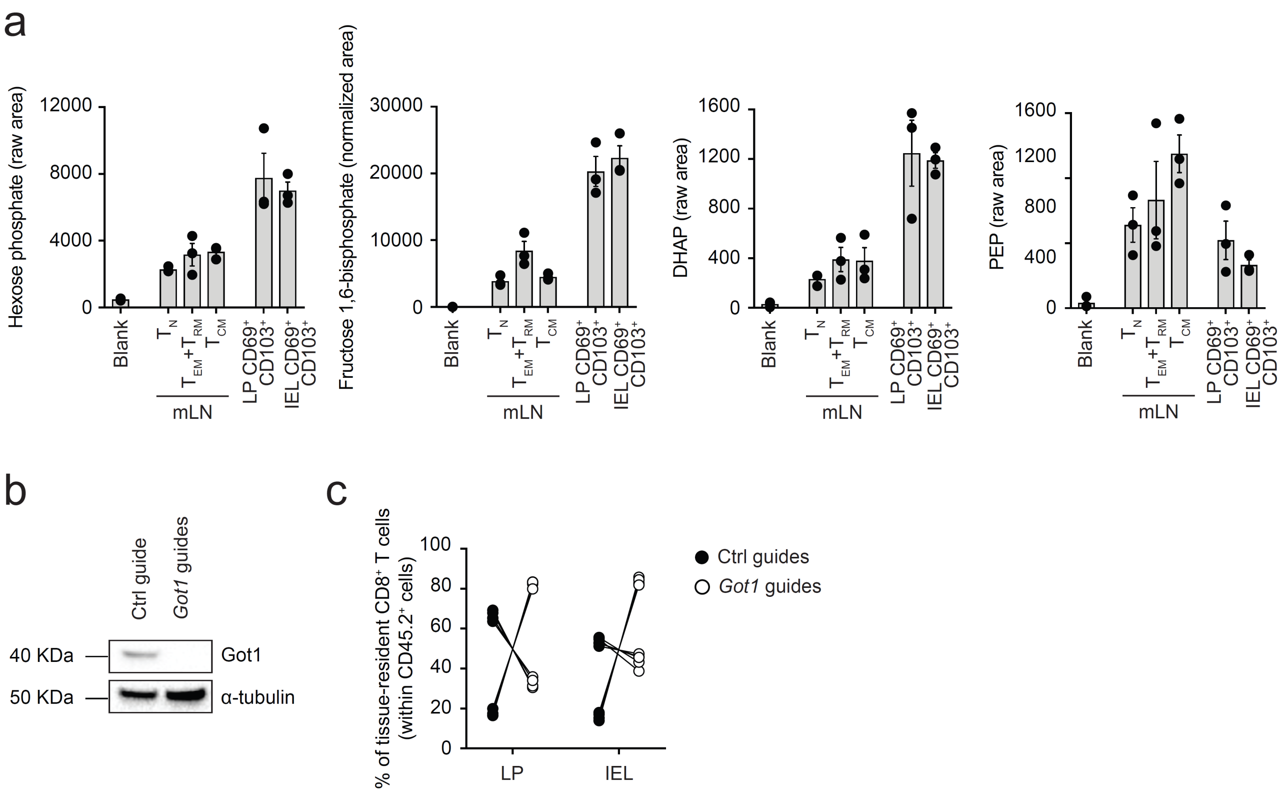


*Supplementary Figure 7. Reduced NAD⁺ impinges on the glycolysis pathway*

*a*. Mass spectrometry quantification of Hexose phosphate, fructose 1,6-bisphosphate, DHAP and PEP in the indicated cell populations. The bars show mean ± SEM. Data show three independent experiments analyzed simultaneously. Statistics were performed using one-way ANOVA and Tukey's multiple comparison correction.

*b*. Western blot analysis of Got-1 in CD8⁺ T cells isolated from C57BL/6J mice, treated for CRISPR-Cas9-mediated deletion of *Got1* and activated in (IL-15/TGF-β)-T cell-polarizing conditions for 6 days. Data are from one experiment.

*c*. Flow cytometry analysis of the distribution of Ctrl vs *Got1*-deleted CD8⁺ T cells within the population of CD45.2⁺ cells upon *LmOVA* challenge in LP and IEL fraction. Lines in the dot plot show pairing within single mice, and the dot plots show cumulative data of n = 9 biological replicates over two independent experiments. Statistics were performed using two-way ANOVA and Sidak's multiple comparison correction.

**Uncropped scans of blots**

*Supplementary Figure 4c*

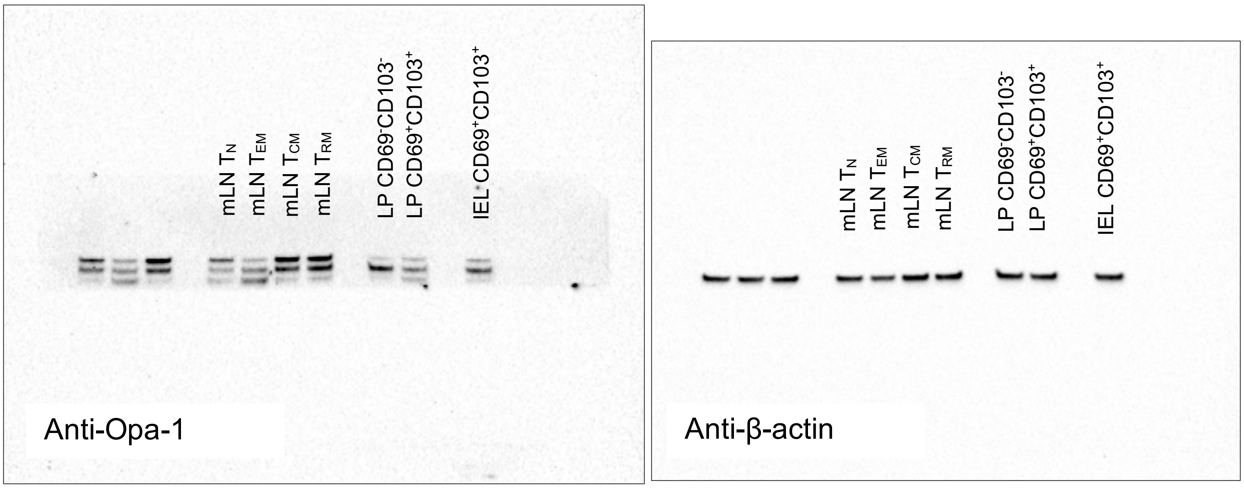


*Supplementary Figure 5i*

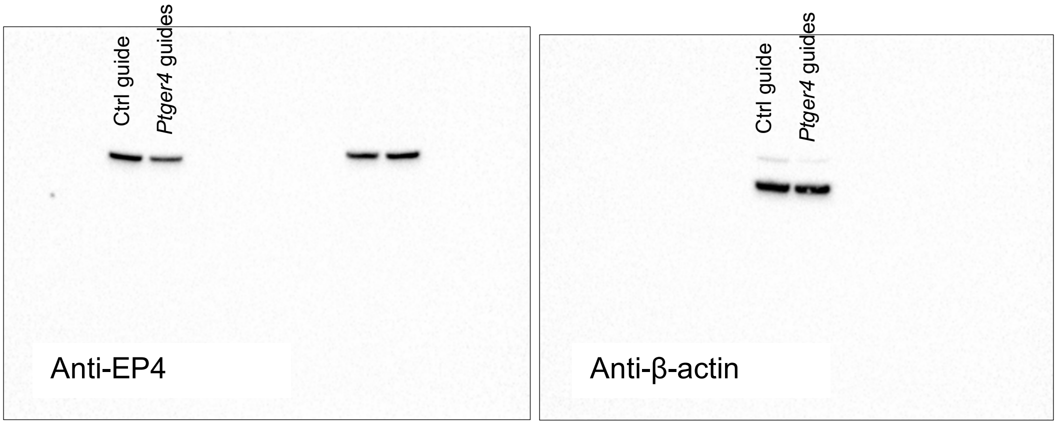


*Supplementary Figure 6e*

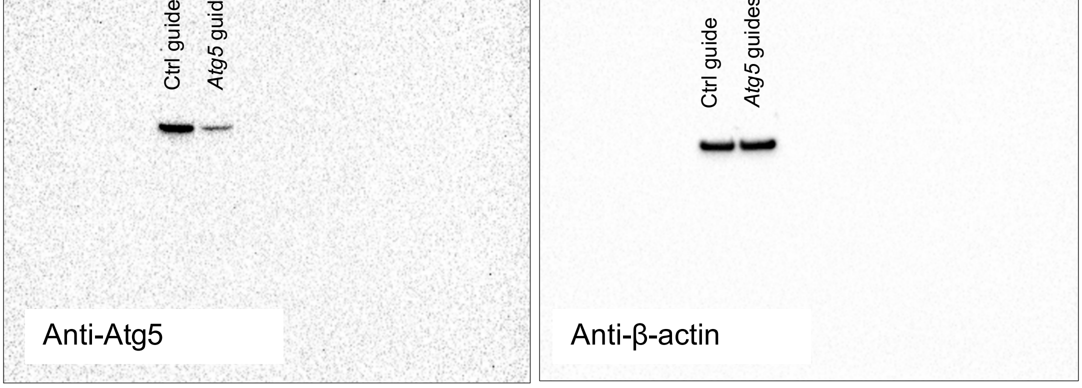

*Supplementary Figure 6i*

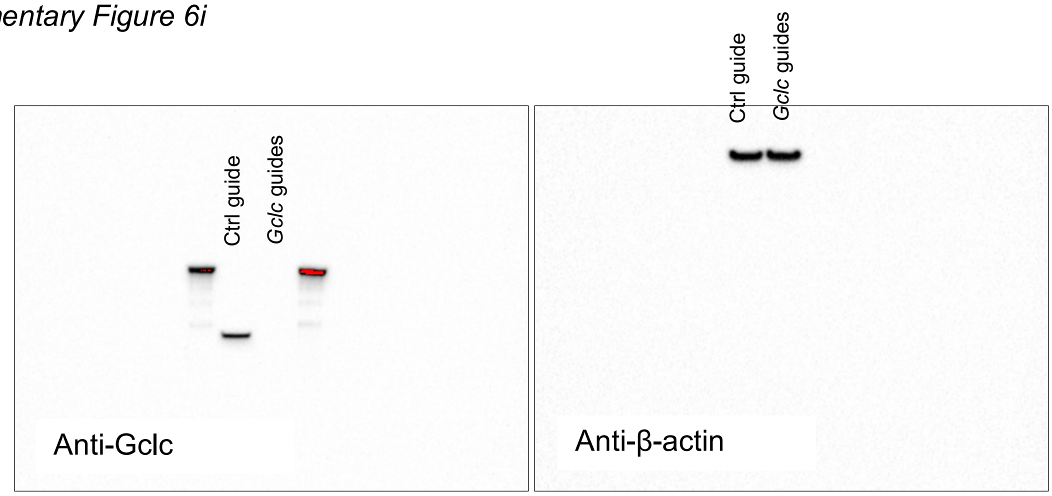


*Supplementary Figure 7b*

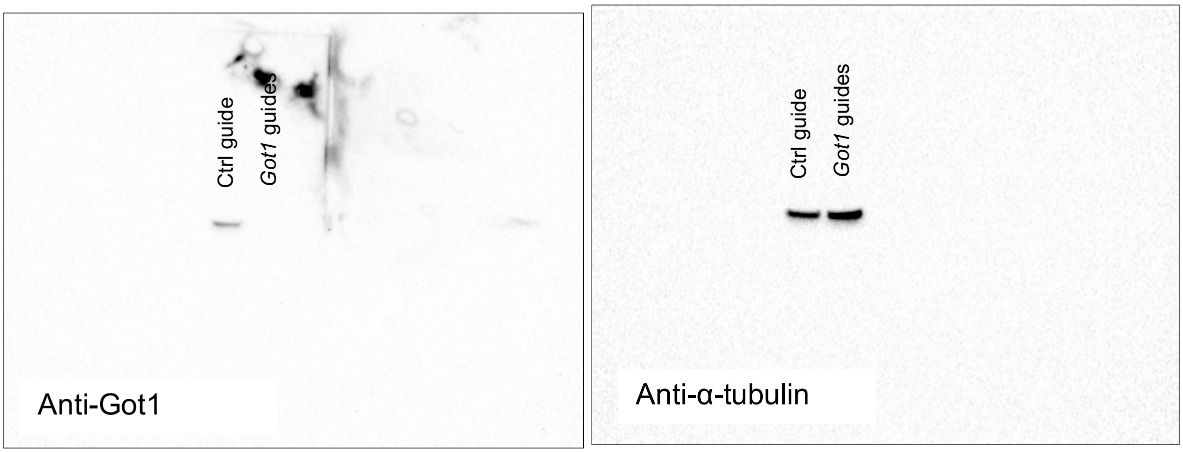

Supplement: Supplementary file 1 — Supplementary Information [file 41467_2024_44689_MOESM1_ESM.pdf]
