# Supplementary material for: Prostaglandin E2 controls the metabolic adaptation of T cells to the intestinal microenvironment
Source: Nat Commun. 2024 Jan 11;15:451. doi: 10.1038/s41467-024-44689-2 (PMC10781727; doi:10.1038/s41467-024-44689-2)
Supplement: Supplementary file 3 — Reporting Summary [file 41467_2024_44689_MOESM3_ESM.pdf]

nature portfolio

Corresponding author(s): Matteo Villa, Erika L. Pearce

Last updated by author(s): 27.11.2023

Reporting Summary

Nature Portfolio wishes to improve the reproducibility of the work that we publish. This form provides structure for consistency and transparency in reporting. For further information on Nature Portfolio policies, see our Editorial Policies and the Editorial Policy Checklist.

Statistics

For all statistical analyses, confirm that the following items are present in the figure legend, table legend, main text, or Methods section.

n/a	Confirmed	
☐	☒	The exact sample size (*n*) for each experimental group/condition, given as a discrete number and unit of measurement
☒	☐	A statement on whether measurements were taken from distinct samples or whether the same sample was measured repeatedly
☐	☒	The statistical test(s) used AND whether they are one- or two-sided<br>*Only common tests should be described solely by name; describe more complex techniques in the Methods section.*
☐	☒	A description of all covariates tested
☐	☒	A description of any assumptions or corrections, such as tests of normality and adjustment for multiple comparisons
☐	☒	A full description of the statistical parameters including central tendency (e.g. means) or other basic estimates (e.g. regression coefficient) AND variation (e.g. standard deviation) or associated estimates of uncertainty (e.g. confidence intervals)
☐	☒	For null hypothesis testing, the test statistic (e.g. *F*, *t*, *r*) with confidence intervals, effect sizes, degrees of freedom and *P* value noted<br>*Give P values as exact values whenever suitable.*
☒	☐	For Bayesian analysis, information on the choice of priors and Markov chain Monte Carlo settings
☒	☐	For hierarchical and complex designs, identification of the appropriate level for tests and full reporting of outcomes
☒	☐	Estimates of effect sizes (e.g. Cohen's *d*, Pearson's *r*), indicating how they were calculated

Our web collection on statistics for biologists contains articles on many of the points above.

Software and code

Policy information about availability of computer code

Data collection

No softwares or codes were used to perform data collection.

Data analysis

For references, use the list provided in the manuscript.

Flow cytometry raw data were analyzed with FlowJo (BD).

Single cell RNA seq:
Samples were demultiplexed and aligned using Cell Ranger 6.1 (10X Genomics) to genome build mm10 to obtain a raw read count matrix of barcodes corresponding to cells and features corresponding to detected genes. Read count matrices were processed, analyzed and visualized in R v. 4 (R Core Team 2013) using Seurat v. 4 [81] with default parameters in all functions, unless specified. Poor quality cells, with low total unique molecular identifier (UMI) counts (< 4,000), low number of detected genes (< 1,500) and high percent mitochondrial gene expression (> 20%), were excluded. Filtered samples were normalized using a regularized negative binomial regression (SCTransform) [82] and merged. Principal component analysis was performed on merged matrices and the first 50 principal components were used for downstream analysis. Integrated gene expression matrices were visualized with a UMAP (McInnes et al. 2018; arXiv:1802.03426) as a dimensionality reduction approach. For ADT libraries, samples were demultiplexed and then processed with CITE-seq-Count v. 1.4.5 (Roelli, P et al. 2019). Later merged and harmonized to remove biological replicate associated effects using harmony [83]. A combined UMAP for RNA and ADT profiles was generated by building a weighted nearest neighbors graph based on their respective PCA projections [81]. Resolution for cell clustering was determined by evaluating hierarchical clustering trees at a range of resolutions (0-1.2) with Clustree [84], selecting a value inducing minimal cluster instability. Differentially expressed genes between clusters were identified as those expressed in at least 25% of cells with a greater than +0.5 log10 fold change and an adjusted p value of less than 0.01, using the FindMarkers function in Seurat v.4 with all other parameters set to default. Ribosomal protein genes were excluded from results. Cluster specific genes were explored for pathway enrichment using

Biological Process Gene Ontology annotation with goseq v. 1.40.0 [85]. Gene set scores were calculated using the AddModuleScore function in Seurat v.4 with default parameters. Briefly, the average expression levels of each identified gene set were calculated on a single cell level and subtracted by the aggregated expression of randomly selected control gene sets. For this purpose, target genes are binned based on averaged expression, and corresponding control genes are randomly selected from each bin. Cell trajectories and pseudotime estimations were calculated with Slingshot v. 1.6.1 [35], using UMAP projection and pre-calculated clustering as input for getLineages and getCurves functions with default parameters, setting origin to cluster 6. Trajectory dependent gene regulation was visualized by fitting general additive models [79] using gam v. 1.20 (Hastie, T 2020) using locally estimated scatterplot smoothing (loess) smooth terms.

Western blot:
Optical density of the signals on the film was quantified using grayscale measurements in ImageJ software (NIH) and converted to fold change, normalized to the loading control.

Fluorescence microscopy:
Confocal images were deconvoluted using Huygens software and analyzed using Imaris imaging software.

Bulk RNAseq:
Sequenced libraries were processed with deepTools [86, 87], using STAR [88], for trimming and mapping, and featureCounts [89] to quantify mapped reads. Raw mapped reads were processed in R (Lucent Technologies) with DESeq2 [90], to determine differentially expressed genes (adjusted p value < 0.05, log2 fold change > 1) and generate normalized read counts to be visualized as heatmaps using Morpheus (Broad Institute).

Metabolomics:
Metabolites were analyzed and identified by fragmentation and retention time using Metaboscape software (Bruker).
Raw data were analyzed using the R package automRm [93]. Further analysis was performed using MassHunter software (Agilent).

Statistical analysis:
Statistical analysis was performed using Graphpad Prism 7 Software. Analysis was performed using Metaboscape software (Bruker).

For manuscripts utilizing custom algorithms or software that are central to the research but not yet described in published literature, software must be made available to editors and reviewers. We strongly encourage code deposition in a community repository (e.g. GitHub). See the Nature Portfolio guidelines for submitting code & software for further information.

Data

Policy information about availability of data

All manuscripts must include a data availability statement. This statement should provide the following information, where applicable:

- Accession codes, unique identifiers, or web links for publicly available datasets
- A description of any restrictions on data availability
- For clinical datasets or third party data, please ensure that the statement adheres to our policy

Data can be found under GEO accession number GSE200568, a SuperSeries containing both the scRNAseq data and bulk RNAseq data.

Research involving human participants, their data, or biological material

Policy information about studies with human participants or human data. See also policy information about sex, gender (identity/presentation), and sexual orientation and race, ethnicity and racism.

Reporting on sex and gender	Samples originated from both sexes have been used to perform our research. Data are not presented disaggregated as samples from both sexes showed comparable results.
Reporting on race, ethnicity, or other socially relevant groupings	Race, ethnicity, or any socially relevant groupings played a role in the recrutiment process. Patients were recruited sequentially as they approached the Universiyt Hospital Freiburg clinic.
Population characteristics	Biopsies from n=5 healthy donors presenting for routine endoscopy were included in the study. None of the included biopsies showed macroscopic signs of active inflammation, and patients were asymptomatic.
Recruitment	Human participants were recruited from the University Hospital Freiburg in a sequential manner with every patient undergoing endoscopy on the days of recruitment being approached for participation in the study. Given no pre-selection based on age, gender, medical history (except for gastrointestinal diseases), or any other criteria, we do not perceive any bias in the recruitment of individuals.
Ethics oversight	Institutional Review Boards (Ethics Committee of the Albert Ludwigs University, Freiburg; #407/16 and #14/17). The study was performed in agreement with the principles expressed in the Declaration of Helsinki (2013).

Note that full information on the approval of the study protocol must also be provided in the manuscript.

Field-specific reporting

Life sciences study design

All studies must disclose on these points even when the disclosure is negative.

Sample size	Sample size for animal experiment was calculate using professional advice and the statistical software G-power. Sample size for single cell RNA and bulk RNA sequencing was chosen according to personal experience (n = 3 biological replicates) and in view of financial constraints. Sample size for human data was chosen according to personal experience and overall availability of samples.
Data exclusions	In the single cell analysis data were curated to exclude obvious contaminant cell populations. No data were excluded elsewhere throughout the manuscript.
Replication	Majority of the experiments were repeated 3 or more times, including the single cell data. Some experiments were repeated only twice or one time, if the outcome was not essential for the validation of our hypothesis. The replicate number is always clearly stated in the legends.
Randomization	No conscious biases were used to assign experimental groups. We thus believe that test samples were randomly assigned.
Blinding	No blinding was used to assign and constitute experimental groups. Our readouts consist of objective measurements (such as flow cytometry and quantification of western blots) that do not require blinding for unbiased data analysis.

Reporting for specific materials, systems and methods

We require information from authors about some types of materials, experimental systems and methods used in many studies. Here, indicate whether each material, system or method listed is relevant to your study. If you are not sure if a list item applies to your research, read the appropriate section before selecting a response.

Materials & experimental systems

n/a	Involved in the study	
☐	☒	Antibodies
☒	☐	Eukaryotic cell lines
☒	☐	Palaeontology and archaeology
☐	☒	Animals and other organisms
☒	☐	Clinical data
☒	☐	Dual use research of concern
☒	☐	Plants

Methods

n/a	Involved in the study	
☒	☐	ChIP-seq
☐	☒	Flow cytometry
☒	☐	MRI-based neuroimaging

Antibodies

Antibodies used

Flow cytometry:
Mouse antibodies:
anti-CD45 (clone 30-F11, 1:200, APC), anti-CD8α (clone 53-6.7, 1:200, BV605), anti-CD8β (clone 53-5.8, 1:200, PE-Cy7), anti-CD62L (clone MEL-14, 1:200, Pacific blue or PerCP-Cy5.5), anti-CD44 (clone IM7, 1:200, APC-Cy7), anti-CD69 (clone H1.2F3, 1:200, PE or BV711), anti-CD64 (clone X54-5/7.1, 1:200, BV605), anti-F4/80 (clone BM8, 1:100, PE-Cy7), anti-Ly6G (clone 1A8, 1:200, PerCP-Cy5.5), anti-TIM4 [clone 54(RMT4-54), 1:200, PE], anti-CD45.2 (clone 104, 1:200, APC), anti-Thy1.1 (clone OX-7, 1:400, BV605) from Biolegend.

anti-CD103 (clone 2E7, 1:200, APC or eFluor 405), from Invitrogen.

anti-Siglec F (clone E50-2440, 1:200, APC), from BD.

Human antibodies:
anti-CD19 (clone HIB19, 1:200, PE or APC-Cy7), anti-CD3 (clone HIT3a, 1:200, PE or APC), anti-CD8 (clone HIT8a, 1:200, Pacific blue), anti-CD69 (clone FN50, 1:200, PE-Cy7), anti-CD103 (clone Ber.ACT8, 1:200, BV711), anti-CD45RA (clone HI100, 1:200, APC), anti-CD27 (clone LG3A10, 1:200, PE-Cy7), from Biolegend.

Intracellular staining:
anti-interferon γ (clone XMG1.2, 1:200, PerCP-Cy5.5), anti-TNF (clone MP6-XT22, 1:200, BV421), from Biolegend.

Single cell sequencing:
TotalSeqTM-A Custom Mouse panel including 198 antibodies was obtained from Biolegend (#99833).

Western blot:
anti-TOM20 (clone D8T4N, 1:1,000, Cell Signaling), anti-TFAM (polyclonal, 1:1,000, Abcam), anti-β actin (clone 13E5, 1:10,000, Cell Signaling), anti-OPA1 (clone 18/OPA-1, 1:2,000, BD), anti-EP4 (encoded by Ptger4, clone C-4, 1:100, Santa Cruz), anti-ATG5 (clone

	D5F5U, 1:1,000, Cell Signaling), anti-Gclc (clone OTI1A3, 1:1,000, Invitrogen), anti-LC3B (polyclonal, 1:3,000, Sigma), anti-SQSTM1/p62 (clone D1Q5S, 1:1,000, Cell Signaling), anti-Got-1 (clone E4A4O, 1:1,000, Cell Signaling).
Validation	Antibodies were all sourced commercially with independent validations and citations.

Animals and other research organisms

Policy information about studies involving animals; ARRIVE guidelines recommended for reporting animal research, and Sex and Gender in Research

Laboratory animals	For references, use the list provided in the manuscript.<br><br>C57BL/6J mice (RRID: IMSR_JAX:000664), PhAM mice (RRID: IMSR_JAX:018397), CD4Cre mice (RRID: IMSR_JAX:017336), Drp1floxed mice (gift from Hiromi Sesaki), Great mice (interferon-γ reporter, RRID: IMSR_JAX:017580), major histocompatibility complex (MHC) class I-restricted ovalbumin (OVA)-specific TCR OT-I transgenic mice (RRID: IMSR_JAX:003831), CD45.1 congenic mice (RRID: IMSR_JAX:002014), Thy1.1 congenic mice (RRID: IMSR_JAX:000406) were purchased from The Jackson Laboratory. Mito-roGFP2-Orp1 mice (tracking mitochondrial H2O2 production) [46, 47] were a kind gift from Tobias P. Dick (DKFZ).<br>All mice were maintained in the animal facilities at the Max Planck Institute for Immunobiology and Epigenetics, under specific-pathogen free (SPF) conditions and following institutional animal use and care guidelines. Mice were exposed to a 14h/10h light/dark cycle and fed ad libitum with acidified water (pH 2.5-3.3). Room temperature was maintained at 21° C, with humidity around 60%. Euthanasia and animal procedures were conducted on 8-12 weeks old male and/or female mice, age-matched and sex-matched.
Wild animals	No wild animals were used in this study.
Reporting on sex	Male and female mice have been used in our research, and clearly stated in the Methods section.
Field-collected samples	No field collected samples were used in this study.
Ethics oversight	All mice were maintained in the animal facilities at the Max Planck Institute for Immunobiology and Epigenetics, under specific-pathogen free (SPF) conditions and following institutional animal use and care guidelines. Euthanasia and animal procedures were conducted in compliance to § 4, paragraph 3 of the German Animal Protection Act, animal licenses 35-9185.81/G-20/107 and 35-9185.81/G-20/101, approved by the Regierungspräsidium Freiburg.

Note that full information on the approval of the study protocol must also be provided in the manuscript.

Plants

Seed stocks	*Report on the source of all seed stocks or other plant material used. If applicable, state the seed stock centre and catalogue number. If plant specimens were collected from the field, describe the collection location, date and sampling procedures.*
Novel plant genotypes	*Describe the methods by which all novel plant genotypes were produced. This includes those generated by transgenic approaches, gene editing, chemical/radiation-based mutagenesis and hybridization. For transgenic lines, describe the transformation method, the number of independent lines analyzed and the generation upon which experiments were performed. For gene-edited lines, describe the editor used, the endogenous sequence targeted for editing, the targeting guide RNA sequence (if applicable) and how the editor was applied.*
Authentication	*Describe any authentication procedures for each seed stock used or novel genotype generated. Describe any experiments used to assess the effect of a mutation and, where applicable, how potential secondary effects (e.g. second site T-DNA insertions, mosiacism, off-target gene editing) were examined.*

Flow Cytometry

Plots

Confirm that:

- [x] The axis labels state the marker and fluorochrome used (e.g. CD4-FITC).
- [x] The axis scales are clearly visible. Include numbers along axes only for bottom left plot of group (a 'group' is an analysis of identical markers).
- [x] All plots are contour plots with outliers or pseudocolor plots.
- [x] A numerical value for number of cells or percentage (with statistics) is provided.

Methodology

Sample preparation	Please, see the Methods section for a detailed description of sample preparation before flow cytometry.
Instrument	Cells were acquired on BD LSRFortessaTM or BD FACSymphonyTM, or they were sorted using BD FACSAriaTM. Raw data were analyzed with FlowJo (BD).
Software	Raw data were analyzed with FlowJo (BD).

Cell population abundance	Cell numbers are stated in the Methods section. Post-sort purity was assessed by re-analyzing the sorted population and was always above 99% pure.
Gating strategy	To analyze the CD8+ T cell response in humans we used the surface markers CD45RA, CD27, CD69 and CD103, identifying by flow cytometry five CD8+ T cell populations: CD45RA+CD27+ TN, CD45RA-CD27- TEM, CD45RA+CD27- TEMRA and CD45RA-CD27+ TCM isolated from blood; CD69+CD103+ TRM isolated from gut biopsies. To analyze the continuum of the gut immune response in mouse we used the surface markers CD62L, CD44, CD69 and CD103, identifying by flow cytometry seven CD8+ T cell populations that approximate the transition of CD8+ T cells from mLN to the intestine: CD62L+CD44- TN, CD62L+CD44+ TCM, CD62L-CD44+CD69-CD103- TEM and CD62L-CD44+CD69+CD103+ TRM isolated from mLN; CD44+CD69-CD103- and CD44+CD69+CD103+ from LP; and CD44+CD69+CD103+ induced CD8ab cells from IEL.

☒ Tick this box to confirm that a figure exemplifying the gating strategy is provided in the Supplementary Information.
